# Near full-length HIV type 1M genomic sequences from Cameroon: Evidence of early diverging under-sampled lineages in the country

**DOI:** 10.1093/emph/eov022

**Published:** 2015-09-09

**Authors:** Marcel Tongo, Jeffrey R. Dorfman, Melissa-Rose Abrahams, Eitel Mpoudi-Ngole, Wendy A. Burgers, Darren P. Martin

**Affiliations:** ^1^International Centre for Genetic Engineering and Biotechnology (ICGEB), Cape Town, South Africa;; ^2^Division of Immunology, Department of Pathology, Faculty of Health Sciences, University of Cape Town, Cape Town, South Africa;; ^3^Division of Medical Virology, Department of Pathology and; ^5^Division of Computational Biology, Department of Integrated Biology Sciences and Institute of Infectious Disease and Molecular Medicine, Faculty of Health Sciences, University of Cape Town, Cape Town, South Africa; and; ^4^Institute of Medical Research and Study of Medicinal plants (IMPM), Yaoundé, Cameroon

**Keywords:** HIV-1 diversity, divergent, Cameroon, recombinant, outlier

## Abstract

**Background:** Cameroon is the country in which HIV-1 group M (HIV-1M) likely originated and is today a major hotspot of HIV-1M genetic diversity. It remains unclear, however, whether the highly divergent HIV-1M lineages found in this country arose during the earliest phases of the global HIV-1M epidemic, or whether they arose more recently as a result of recombination events between globally circulating HIV-1M lineages.

**Methodology:** To differentiate between these two possibilities, we performed phylogenetic analyses of the near full genome sequences of nine newly sequenced divergent HIV-1M isolates and 15 previously identified, apparently unique recombinant forms (URFs) from Cameroon.

**Results:** Although two of the new genome sequences were clearly classifiable within subtype G, the remaining seven were highly divergent and phylogenetically branched either outside of, or very near the bases of clades containing the well characterised globally circulating viral lineages that they were most closely related to. Recombination analyses further revealed that these divergent viruses were likely complex URFs. We show, however that substantial portions (>1 Kb) of three of the new genome sequences and 15 of the previously characterised Cameroonian URFs have apparently been derived from divergent parental viruses that branch phylogenetically near the bases of the major HIV-1M clades.

**Conclusions and implications:** Our analyses indicate the presence in Cameroon of contemporary descendants of numerous early-diverging HIV-1M lineages. Further efforts to sample and sequence viruses from such lineages could be crucial both for retracing the earliest evolutionary steps during the emergence of HIV-1M in humans, and accurately reconstructing the ancestral sequences of the major globally circulating HIV-1M lineages.

## INTRODUCTION

HIV-1 group M (HIV-1M), the group of viruses that are responsible for the global AIDS epidemic [1] consists of nine major lineages called subtypes A–D, F–H, J and K. These lineages are highly diverse and present a major obstacle both to the development of a globally effective HIV-1M vaccine, and to the sustainability of drug treatment [2–6]. The development of interventions to combat HIV-1M and efforts to trace the early evolution of HIV-1M are further complicated by the frequent occurrence of viral variants that have arisen as a result of recombination between two or more HIV-1M subtypes [7]. Besides a multitude of unique recombinant forms (URFs) that have only ever been found infecting single individuals, the Los Alamos National Laboratory (LANL) HIV database (http://hiv-web.lanl.gov/content/hiv-db) presently also recognises 72 circulating recombinant forms (CRFs) that have each been identified in at least three epidemiologically unlinked individuals.

Based on phylogenetic analyses, it is now well accepted that HIV-1M originated from the transmission into humans of a chimpanzee-infecting simian immunodeficiency virus somewhere in southern Cameroon [8–10]. Possibly as a consequence of this, Cameroon is today a hotspot of HIV-1M genetic diversity, with all known HIV-1M subtypes, at least eleven CRFs, and numerous URFs having been found infecting people within this country [11–16]. In the most extensive Cameroonian HIV-1M diversity study to date, Carr *et al.* [17] sampled viruses in rural villages and demonstrated that while ∼90% of isolates could be classified into established subtypes or CRFs (with CRF02_AG accounting for ∼66% of the analysed infections), the remaining unclassifiable isolates were possibly URFs.

Besides being the likely ‘ground-zero’ of the HIV-1M epidemic [17], Cameroon is also the likely origin of the HIV-1O and HIV-1P epidemics; gorillas in the south east of the country have recently been identified as the likely original source of these viruses [18].

Continuously sampling and sequencing HIV-1M isolates in a diversity hotspot such as Cameroon is crucial for epidemiological tracking, informing vaccine development and the accurate reconstruction of the early evolutionary history of HIV-1M. However, classifying an unknown HIV-1 sequence or determining the origins of a presumed CRF/URF is particularly challenging in a region where so many divergent ‘pure’ subtype (i.e. non-inter-subtype recombinant) and inter-subtype recombinant lineages are co-circulating. Compounding the problem is the possibility that over 4% of publically available HIV-1M sequences have been classified incorrectly [19, 20].

Phylogenetic analyses have been widely used to determine the subtype of newly determined HIV-1 sequences; in these analyses, subtype determination is based on the clustering of the new sequence in a phylogenetic tree together with reference sequences of known pure subtypes and CRFs. A key consideration in these analyses is the choice of known sequences - called reference sequences - to serve as representatives of known subtypes and/or CRFs. This choice is, for the most part, *ad hoc* and only rarely, are steps taken to ensure that references are representative of the overall diversity that exists within a specific-known subtype or CRF grouping. Crucially, biases in the choice of reference sequences could result in the misclassification of new sequences. Also, it is often not appreciated that simply because a new sequence is most closely related to sequences belonging to a particular known subtype, it does not necessarily mean that the new sequence should be classified as belonging to that subtype. Specifically, rather than clustering phylogenetically amongst the sequences of that subtype, the sequence might fall on a ‘basal’ lineage that branches before the most recent common ancestor (MRCA) of all the known sequences of the subtype. Whereas in the former case the sequence should be classified as belonging to the subtype, in the latter case it might be better to leave the sequence unclassified.

The difficulties inherent in classifying the numerous divergent Cameroonian HIV-1M isolates discovered thus far have hampered our understanding of how these viruses fit into the established HIV-1M taxonomy. It therefore remains unclear whether these viruses are simply recently arising URFs, or whether they are the contemporary descendants of rare early-diverging HIV-1M lineages that merely failed to disseminate globally. In an effort to explore these two possibilities we sequenced and thoroughly analysed nine near full-length HIV-1 genome sequences that, based on *gag* and *nef* sequencing [16], had been previously identified as potentially belonging to such lineages. We also analysed fifteen published near full-length divergent Cameroonian HIV-1M genomes that had previously been identified as likely URFs [17].

## METHODOLOGY

### Study participants

Participants were chosen from a total of 64 anonymous blood donors found to be HIV-infected, in a study approved by the Ethics Committees of the Cameroonian Ministry of Health and the University of Cape Town and these participants were described in [16].

### Sequencing and phylogenetic analysis

Based on *gag* and *nef* sequences, we previously identified nine individuals infected with inter-subtype recombinant and/or divergent HIV-1 strains [16]. RNA from plasma samples was manually extracted using the QIAamp Viral RNA Mini Kit (QIAGEN, Hilden, Germany). Nearly full genome length HIV cDNA was generated and amplified using the Thermoscript^TM^ RT-PCR system (Invitrogen, Carlsbad, USA). DNA amplification was performed using the single genome amplification technique [21] with the Expand Long Template PCR System (Roche, Mannheim, Germany). The PCR primers and protocol used are described in reference [22]. This technique involved cDNA template dilution (eight replicate reactions were prepared per dilution) and direct sequencing of near full-length amplicons obtained from dilutions yielding <30% positive reactions. This technique serves to ensure amplification from a single template and elimination of template recombination during PCR. In some cases, multiple second round PCR amplifications were combined to provide sufficient template for sequencing. Sequenced fragments were assembled using ChromasPro (http://technelysium.com.au/?page_id=27).

Coding sequences were generated and aligned using MUSCLE with manual editing in IMPALE (http://www.cbio.uct.ac.za/∼arjun/), together with a representative selection of near full-length sequences from all published subtypes, CRFs and sequences labelled as ‘U’ that were available in the LANL (http://hiv-web.lanl.gov/content/hiv-db) database in June 2014. The representative sequences were specifically selected to include the broadest diversity of sequences previously identified as belonging to known HIV-1M subtypes and CRFs. This was achieved by constructing maximum likelihood (ML) trees from all available full-length sequences for each subtype and CRF using Fastree [23] implemented in RDP4 [24], and selecting one sequence from each of the up to 20 most basal lineages from the root of these subtypes and CRFs (Supplementary Fig. S1A). This approach to selecting subtype and CRF reference sequences ensured both that these sequences represented the broadest diversity of known HIV lineages, and that we did not include too many superfluous sequences in our subsequent analyses. A ML phylogenetic tree was constructed from these sequences with 500 full ML boostrap replicates using RAxML version 8 [25] implemented in CIPRES [26]. The tree was midpoint rooted.

Divergent sequences were defined as either (i) those residing on isolated branches outside of sub-trees containing previously defined HIV-1 subtype or CRF lineages, or (ii) those clustering with low degrees of associated bootstrap support within subtrees containing previously defined HIV-1 subtype or CRF lineages. Importantly, although the uncertain phylogenetic placement of this latter group of divergent sequences might be due to their being descended from a lineage that diverged close to the origin of their associated subtype or CRF lineages, it is not possible to rule out that these sequences are in fact genuinely clustered within these subtype lineages (Supplementary Fig. S1B).

### Recombination analysis

Inter-subtype recombination events were inferred using the booscanning method [27] implemented in Simplot [28]. The newly sequenced and previously described viruses were queried against isolates from subtypes A to D, F to H, J, K, CRF01_AE and CRF02_AG and in some cases CRF lineages that they were identified as being most closely related to in the ML tree. The reliability of plot topologies was assessed by bootstrapping with 500 replicates, and a sliding window of 500 bp advancing with 50-bp increments. A 90% consensus of lineages of each clade was also used. Segments were assigned to a particular clade when its peak was above 70% bootstrap support.

Nucleotide sequences were deposited in GenBank [KR017771-KR017779].

## RESULTS

### Discovery of new highly divergent Cameroonian HIV-1M lineages

Based on the results of *gag* and *nef* phylogenetic analyses of Cameroonian HIV-1M isolates carried out in a previous study [16], nine viruses were selected for full-length genome sequencing. The isolates selected for near full-genome sequencing were chosen either because in this earlier study they were determined to be divergent sequences that phylogenetically clustered on isolated/basal branches of the known subtypes or CRF clades that they were most closely related to, or appeared to have *nef* and *gag* genes that clustered with different groups of sequences (i.e. they were potentially recombinants). We obtained near full-length sequences for eight of the nine isolates but only sequenced 78% of the KR017775 genome; all attempts to obtain sequences from the *pol* region of this genome failed.

**Figure 1.**
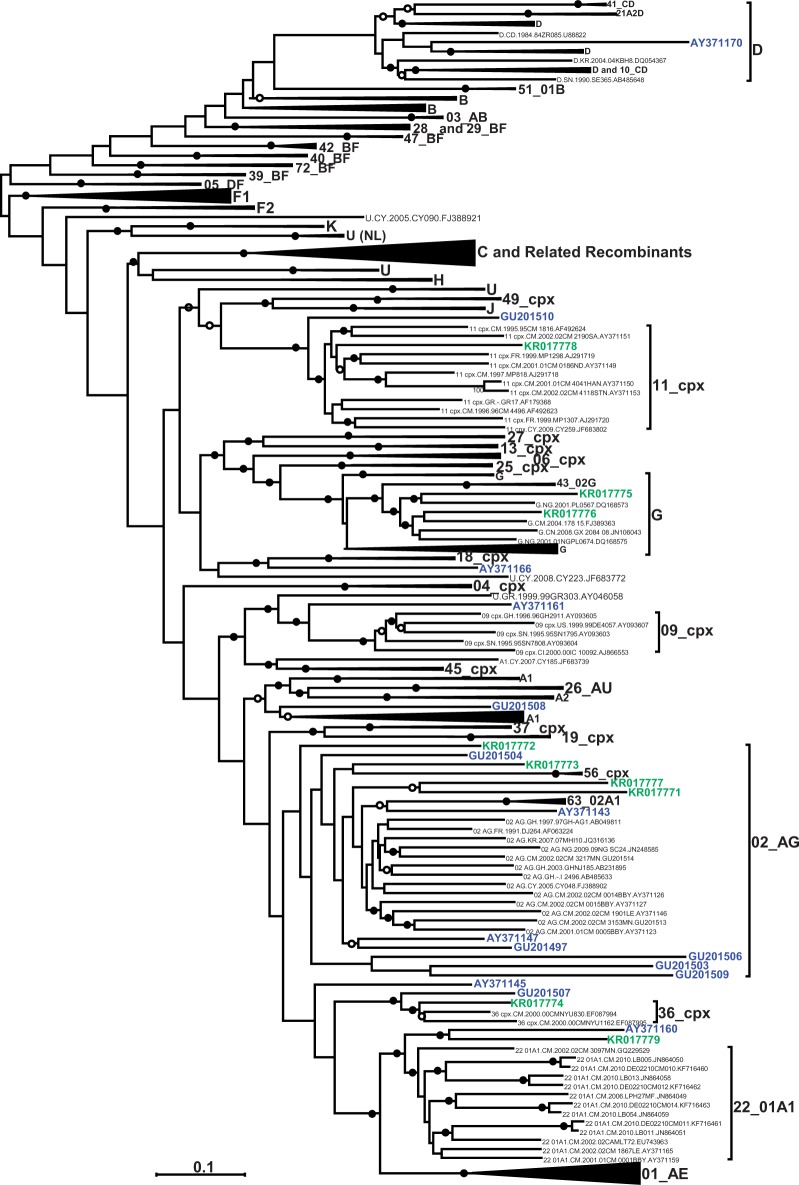
Maximum likelihood tree indicating the phylogenetic placement of the new near full-length genome sequences characterised in this study (in green) plus 15 other previously identified divergent HIV-1M genomes from Cameroon (in blue) and a representative selection of near full-length sequences from all published subtypes, CRFs and unclassified sequences available in the LANL database (http://hiv-web.lanl.gov/content/hiv-db) in June 2014. Some clades have been collapsed for the sake of clarity. A larger tree with un-collapsed clades is available on request. The tree was constructed with 500 full ML bootstrap replicates using RAxML. Solid and open circles indicate branches with >70 and 50% bootstrap support, respectively. The tree was midpoint rooted

The tree in Figure 1 shows the phylogeny of the newly sequenced full length Cameroonian samples. However, this tree is not an appropriate representation of evolutionary history of these sequences due to the inclusion of recombinationally derived genome fragments within many of the represented sequences. Based on previous phylogenetic analyses of the *gag* and *nef* genes of these isolates, it was apparent that sequences from patients BS57 (KR017778), BS29 (KR017773), BS40 (KR017774) and BS72 (KR017779) grouped at the base of the clades with which they were most closely related [16]. The near full-length sequences from these patients also branched close the base of the subtype or CRF clades that they were most closely related to (indicated in green in Fig. 1): the phylogenetic placement of KR017778 is uncertain and it too possibly branches near the base of the CRF11_cpx sub-tree; the KR017773 sequence branched at the base of a sub-tree that included all CRF02_AG lineages and the related recombinant, CRF63_02A1; the KR017774 sequence was an outlier of the CRF36_cpx clade; the KR017779 sequence and a previously described sequence, AY371160, clustered at the base of the CRF22_01A1 sub-tree; and the KR017772 sequence branched basal to CRF02_AG and all its close relatives.

The sequences from patients BS11 (KR017771) and BS55 (KR017777), which were both previously inferred to be possible inter-subtype recombinants [16], formed a deep branch together with a previously characterised sequence AY371143, at the base of the CRF02_AG subtree. This suggests that these sequences might indeed be recombinants with a predominantly CRF02_AG origin (indicated in green in Fig. 1).

Finally, despite the *gag* sequences from BS46 (KR017775) and BS48 (KR017776) branching near the base of the subtype G sub-tree [16], the near full-length genome sequences from these patients clustered well within the subtype G sub-tree (indicated by green sequences in Fig. 1).

All the previously characterised Cameroonian URF sequences [17] included in this analysis were highly divergent at the full-genome scale, phylogenetically branching either outside of, or at the base of clades that they were most closely related to (indicated in blue in Fig. 1).

### The new highly divergent Cameroonian sequences are likely URFs

To test whether the phylogenetic placement of some of the new divergent full-length genome sequences on the outskirts of known clades was a consequence of their being recombinant, we tested these sequences for evidence of inter-subtype recombination events using the bootscanning method [27].

**Figure 2.**
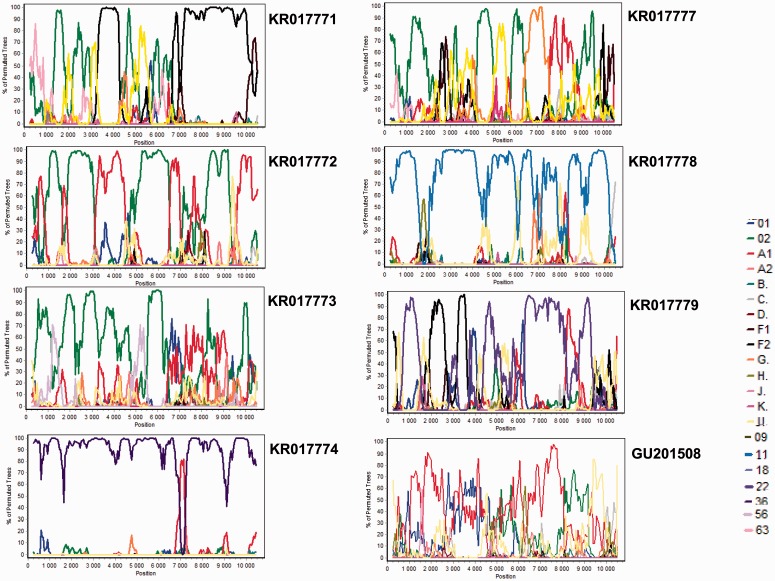
Recombination analysis of the newly sequenced strains plus a previously described strain GU201508. The multiple genome alignment used to calculate the consensus reference sequences (90% threshold) included the same references as used for the phylogenetic tree analysis. The newly sequenced and previously described viruses were queried against strains from subtypes A to D, F to H, J, K, CRF01_AE, and CRF02_AG and, in some cases, lineages of viruses they were most closely related to in the ML tree; the reliability of plot topologies was assessed by bootstrapping with 500 replicates, and a sliding window of 500 bp advancing with 50-bp increments

KR017778 was queried against all the standard reference lineages (A to D, F to H, J, K, 01_AE and 02_AG) and CRF11_cpx as its phylogenetic placement within the CRF11-cpx sub-tree had 9% associated bootstrap support. The bootscan plot of this virus indicated that, with the exception of three small regions with low degrees of bootstrap support (<70%) that could not properly be associated with any other HIV-1M subtype, the remaining sequence was derived from a parental virus that was most closely related to CRF11_cpx (Fig. 2).

Similarly, KR017774 was scanned against the standard reference isolates and CRF36_cpx (the CRF it was most closely related to) and is apparently an inter-subtype recombinant variant containing sequence fragments derived from parental viruses that were most closely related to CRF36_cpx and A1 (although this subtype is also known to be the parental sequence of all known CRF36_cpx sequences at this position, Fig. 2).

KR017779, which in addition to the standard reference isolates was also scanned against CRF22_01A1, showed a complex recombinant structure with as many as four parental lineages including CRF22_01A1, F2, A1 and an unknown lineage (Fig. 2).

KR017773, queried against the standard reference isolates and CRF56_cpx was clearly most closely related to CRF02_AG until position ∼6400 in the alignment. Different regions of the remainder of the KR017773 genome are most closely related to CRF01_AE, A1, an unknown lineage and CRF02_AG, suggesting that it is potentially a complex URF with as many as four parental lineages (Fig. 2).

KR017771 and KR017777 were scanned against the standard reference isolates and CRF63_02A1 and both showed a complex recombinant structure: CRF63_02A1, CRF02_AG, F2, U and an unknown isolate for KR017771, and CRF02_AG, F2, A1, G, D and U for KR017777 (Fig. 2).

Finally, KR017772 was queried only against the standard references and is apparently a recombinant of CRF02_AG, A1 and an unknown lineage (Fig. 2).

The most interesting observation from similar bootscan analyses performed on 15 previously described Cameroonian URF sequences (Supplementary Fig. S2 except for GU201508) was made with GU201508. This sequence was scanned against the standard reference isolates and, except for small fragments of A1, the rest of the genome could not be convincingly associated with any other HIV-1M subtype (Fig. 2). This suggests either the existence of undiscovered parental sequences, or that this divergent sequence might simply display no obvious evidence of inter-subtype recombination.

### Evidence of highly divergent sequence fragments within Cameroonian URFs

We removed the 22 Cameroonian URFs (the seven newly sequenced ones and the 15 previously described) from our HIV-1M sequence alignment and then split each of these 22 sequences into their constituent recombinationally derived fragments based on the locations of breakpoints inferred during the bootscan analyses. Thirty eight individual recombinationally derived fragments larger than 1000 nucleotides (nt) in length (obtained from 22 of the Cameroonian URFs) were then re-added to the alignment with gap characters being added to the 3′ and 5′ ends of the fragments to ensure that they remained correctly aligned with the remainder of the data set. A ML phylogenetic tree was constructed from the resulting alignment using RAxML [25] treating these gap characters as missing data.

**Figure 3.**
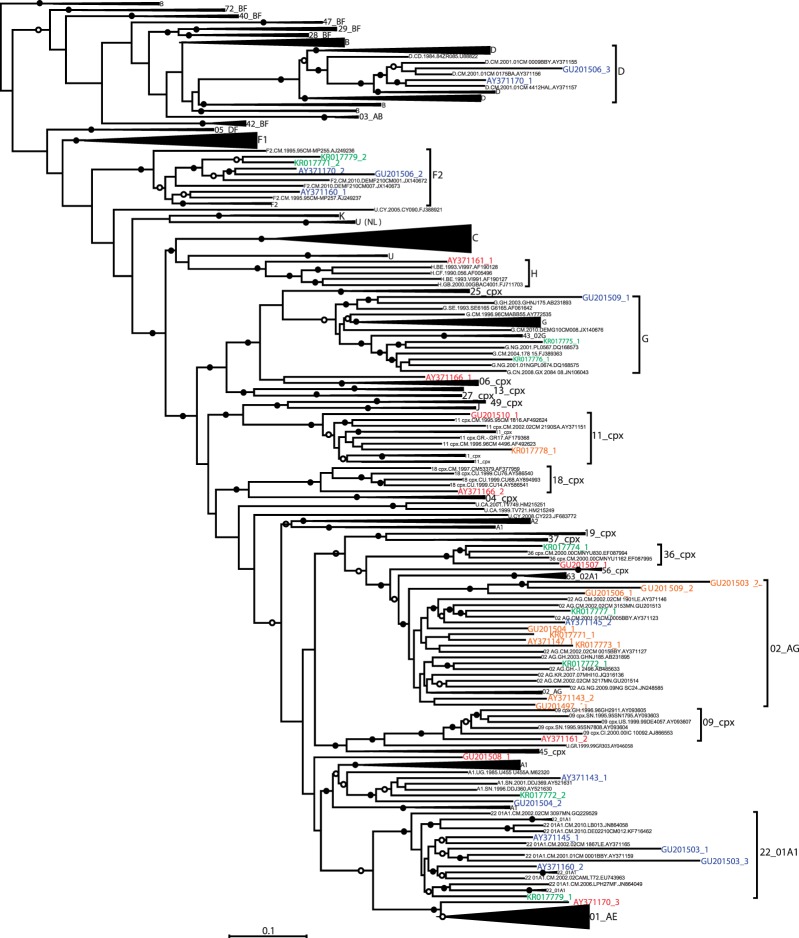
Maximum likelihood tree indicating the phylogenetic placement of recombinationally derived genome fragments drawn from 22 Cameroonian URF sequences in relation to a representative selection of near full-length sequences selected from amongst all published subtype, CRF and unclassified sequences available in the LANL database (http://hiv-web.lanl.gov/content/hiv-db) in June 2014. Some of the clades were collapsed for the sake of clarity. A version of the tree with uncollapsed clades is available on request. The tree was constructed with 500 full ML bootstrap replicates using RAxML. Solid and open circles indicate branches with >70 and 50% bootstrap support, respectively. The tree was midpoint rooted. Orange segments represent divergent sequences residing very near the base of branches of subtrees containing previously defined HIV-1 subtype or CRF lineages whereas red sequences represent divergent sequences residing on isolated branches outside of subtrees containing previously defined HIV-1 subtype or CRF lineages. Green and blue sequences represent recombinationally derived sequence fragments respectively drawn from the newly characterised and previously described Cameroonian URFs that are embedded within well characterised subtype and CRF clades

In this tree, the 3041 nt fragment of KR017773 (KR017773_1) that had apparently been derived from a CRF02_AG like parental sequence clustered with low degree of associated bootstrap support (33%) within the CRF02_AG clade. This might suggests that the parental virus of this sequence diverged very early after the diversification of CRF02_AG (indicated by italicized text in Table 1 and in orange in Fig. 3). The phylogenetic placement of the 7313 nt fragment of KR017778 (KR017778_1) was similarly uncertain with the sequence clustering with a low degree of associated bootstrap support (48%) within the CRF11_cpx clade. The 1248 nt fragment of KR017771 (KR017771_1) that most closely resembled CRF02_AG in the bootscan analysis also clustered with a low degree of bootstrap support (30%) within the CRF02_AG clade (indicated by italicized text in Table 1 and in orange in Fig. 3).

**Table 1.** Detailed representation of all the 38 fragments derived from the 22 Cameroonian URFs

Sample ID	Fragment (nucleotides)	Position
AY371143	AY371143_1 (1200)	Embedded in A1
	AY371143_2 (2411)	*Near the base of CRF02_AG*
AY371145	AY371145_1 (2578)	Embedded in CRF22_01A1
	AY371145_2 (3118)	Embedded in CRF02_AG
AY371147	AY371147_1 (6189)	*Near the base of CRF02_AG*
AY371160	AY371160_1 (1779)	Embedded in F2
	AY371160_2 (4275)	Embedded in CRF22_01A1
AY371161	AY371161_1 (2222)	**Outside of H**
	AY371161_2 (3652)	**Outside of 09_cpx**
AY371166	AY371166_1 (2058)	**Outside of 06_cpx**
	AY371166_2 (3307)	**Outside of 18_cpx**
AY371170	AY371170_1 (2317)	Embedded in D
	AY371170_2 (1328)	Embedded in F2
	AY371170_3 (1796)	**Outside of CRF01_AE**
GU201497	GU201497_1 (5592)	*Near the base of CRF02_AG*
GU201503	GU201503_1 (1787)	Embedded in CRF22_01A1
	GU201503_2 (2943)	**At the base of CRF02_AG**
	GU201503_3 (1501)	Embedded in CRF22_01A1
GU201504	GU201504_1 (4775)	*Near the base of CRF02_AG*
	GU201504_2 (982)	Embedded in A1
GU201506	GU201506_1 (2203)	**At the base of CRF02_AG**
	GU201506_2 (2529)	Embedded in F2
	GU201506_3 (1324)	Embedded in D
GU201507	GU201507_1 (7653)	**Outside of CRF36_cpx**
GU201508	GU201508_1 (3067)	**Did not cluster with a known clade**
GU201509	GU201509_1 (3052)	Embedded in G
	GU201509_2 (3427)	**At the base of CRF02_AG**
GU201510	GU201510_1 (6322)	**Outside of CRF11_cpx**
KR017771^a^	KR017771_1 (1248)	*Near the base of CRF02_AG*
	KR017771_2 (3224)	Embedded in F2
KR017772^a^	KR017772_1 (3556)	Embedded in CRF02_AG
	KR017772_2 (2138)	Embedded in A1
KR017773^a^	KR017773_1 (3041)	*Near the base of CRF02_AG*
KR017774^a^	KR017774_1 (7899)	Embedded in CRF36_cpx
KR017777^a^	KR017777_1 (2301)	Embedded in CRF02_AG
KR017778^a^	KR017778_1 (7313)	*Near the base of CRF11_cpx*
KR017779^a^	KR017779_1 (3562)	Embedded in CRF22_01A1
	KR017779_2 (1101)	Embedded in F2

^a^Newly sequenced Cameroonian viruses.

Furthermore, seven out of 10 of the analysed genome fragments from the newly sequenced Cameroonian URFs clustered within known clades and have therefore likely originated from relatively recent recombination events (i.e. after the diversification of the main subtype lineages) between viruses within well sampled and widely circulating HIV-1M lineages (plain text in Table 1 and green in Fig. 3).

Eleven of the analysed fragments (ranging in size from 1779 to 7653 nt) of the previously characterised Cameroonian URFs, branched outside or at the base of known clades (indicated by bold text in Table 1). Four fragments (ranging in size from 2411 to 6189 nt) clustered with low degrees of bootstrap support (30%) within taxonomically defined clades (indicated by italicised text in Table 1 and orange in Fig. 3), and the remaining 13 fragments branched with ≥70% support within such clades (indicated by plain text in Table 1 and in blue in Fig. 3).

Amongst the sequences that were outliers of known clades, a 2058 nt (AY371166_1) and a 3307 nt (AY371166_2) fragment of AY371166, respectively branched outside of the CRF06_cpx and CRF18_cpx clades. Similarly, a 2222 nt (AY371161_1) and a 3652 nt (AY371161_2) fragment of AY371161, respectively, branched outside of the H and CRF09_cpx clades. Although this may indicate that AY371166 and AY371161 are potentially recombinants of early diverging HIV-1M lineages (indicated by bold text in Table 1 and red in Fig. 3), with the available data it is not possible to determine when the recombination events occurred for these viruses.

Examples of viruses containing genome fragments that branched at the base of known clades included GU201507 with a 7653 nt fragment (GU201507_1) that branched at the base of the CRF36_cpx lineage and 2203–3427 nt segments of GU201503 (GU201503_2), GU201506 (GU201506_1) and GU201509 (GU201509_2) that together formed a deep branch at the base of the CRF02_AG clade. The existence of these latter three fragments within contemporary HIV-1M variants suggests that the source of the fragments is likely a divergent CRF02_AG like lineage which is still circulating (Fig. 3).

Of the several viruses containing fragments of recombinationally derived sequences that phylogenetically branch either outside of, or at the bases of the known subtype or CRF clades (indicated in red in Fig. 3), GU201508 is particularly interesting in that it contains a 3067 nt fragment (GU201508_1) of highly divergent sequence that does not cluster closely with any of the classified HIV-1M clades.

## DISCUSSION

Our characterization of 9 new and 15 previously described phylogenetically divergent near full-length Cameroonian HIV-1M genome sequences significantly increases the breadth of known HIV-1M genetic diversity at the geographical origin of the global HIV-1 epidemic. Inter-subtype recombination has frequently been invoked to explain the branching of such divergent lineages amongst the basal branches of HIV-1M phylogenetic trees [15, 17, 29]. For example, Carr *et al.* [17] identified several sequences, including the ones characterised here, that were outliers of various HIV-1-M clades, and presented analyses indicating that many of these viruses were likely complex URFs: a factor these authors suggested might explain the phylogenetic placement of these divergent lineages.

Although all of the divergent near full-length genome sequences that we analysed here displayed some evidence of inter-subtype recombination, 17/24 of these genomes contained ≥1 Kb of sequence that has apparently been obtained through recombination from highly divergent parental virus lineages. As a consequence, the genome regions derived from these divergent parental viruses were phylogenetically situated outside of all known pure subtype and CRF clades.

The placement of these sequences cannot be attributed to an inadequate choice of representative lineages from each the established subtype and CRF clades, because the representative sequences that we analysed were specifically selected to include the deepest branching sub-lineages of each of these clades. We therefore conclude that these divergent genome fragments are likely genuinely representative of rare, early diverging HIV-1M lineages.

This therefore suggests that at least some portions of the genomes of divergent Cameroonian viruses previously identified as URFs might not in fact be phylogenetically misplaced inter-subtype recombinants. We instead suggest that they are, at least in part, the extant, under-sampled descendants of early diverging HIV-1M lineages. Our hypothesis is that these lineages, or their parental sequences, have simply not undergone the same explosive spread as some of the other HIV-1M lineages from this region. We speculate that the low frequencies of these divergent lineages might be due to their having lower degrees of pathogenicity compared with globally circulating lineages. Alternatively, it is possible that members of these lineages were not carried to locations such as Kinshasa which provided opportunities for high rates of transmission and long distance international movement [30]. An old mosaic lineage was isolated in 1976 in the DRC [31], suggesting that complex recombinant viruses had been circulating in that country before or just after the start of the epidemic. It is therefore also possible that these lineages could have migrated from the DRC to Cameroon. Old lineages have been described in Cameroon and in the Congo basin region in general; CRF36_cpx and CRF37_cpx [32, 33], identified for the first time in remote areas of Cameroon, have been found to contain fragments derived from what appear to be early-diverging subtype A and G variants. This implies that the contemporary descendants of other early-diverging HIV-1M ‘pure-subtype’ lineages may also still be present in the country. The plausibility of this is supported by the fact that a viral lineage such as CRF27_cpx, which is circulating in the DRC at a very low prevalence, has also likely been circulating in equatorial West Africa since the start of the epidemic [34].

It is also worth noting that the majority of the divergent genome fragments that we have identified come from viruses that were sampled in remote rural areas of Cameroon. This is consistent with the hypothesis that, following the primary transmission of the progenitor HIV-1M virus into humans, the first HIV-1M variants arose and intensively diversified in and around the location where the zoonosis occurred [35].

Another striking observation in our study is the number of divergent fragments within the analysed genomes that, although clearly closely related CRF02_AG did not cluster with a high degree of phylogenetic support within the CRF02_AG subtree. This is an indication that the CRF02_AG clade is likely more diverse than previously thought, a factor suggesting that it has likely been present in Cameroon and the surrounding regions since very early in the epidemic. More importantly, the identification of a deep branch with three sequences at the base of the CRF02_AG clade suggests that some of these divergent CRF02_AG lineages are likely still circulating. Ultimately, these and other undiscovered divergent CRF02_AG lineages could be very useful in resolving the controversy surrounding the origin of this clade [19, 36, 37]

## CONCLUSIONS AND IMPLICATIONS

Contemporary divergent HIV-1M genomic sequences could be particularly useful in efforts to reconstruct the early evolutionary history of HIV-1M. These sequences can be preserved within rare entirely inter-subtype recombination-free genomes, or fragmentally dispersed amongst relatively more common inter-subtype recombinants. In addition, these ‘early diverging’ genome sequence fragments will vastly increase the accuracy with which key ancestral HIV-1M sequences (such as the MRCAs of the major subtypes) can be computationally inferred. Besides the utility of such ancestral sequences in retracing the evolutionary steps that HIV-1M took on its path to emergence as a major human pathogen, re-synthesis and experimental characterisation of these ancestral genomes could be used to directly test competing hypotheses relating to the specific biological characteristics of HIV-1M that enabled its emergence and spread.

## SUPPLEMENTARY DATA

Supplementary data is available at *EMPH* online.

Supplementary Data
