# Supplementary material for: Near full-length HIV type 1M genomic sequences from Cameroon: Evidence of early diverging under-sampled lineages in the country
Source: Evol Med Public Health. 2015 Sep 9;2015(1):254–65. doi: 10.1093/emph/eov022 (PMC4600344; doi:10.1093/emph/eov022)
Supplement: Supplementary Data [file supp_2015_1_254__index.html]

Near full-length HIV type 1M genomic sequences from Cameroon — Supplementary Data 

# Near full-length HIV type 1M genomic sequences from Cameroon

## Supplementary Data

files

- Supplementary Data - docx file
- Supplementary Data - pptx file
