# Supplementary material for: Near full-length HIV type 1M genomic sequences from Cameroon: Evidence of early diverging under-sampled lineages in the country
Source: Evol Med Public Health. 2015 Sep 9;2015(1):254–65. doi: 10.1093/emph/eov022 (PMC4600344; doi:10.1093/emph/eov022)
Supplement: Supplementary Data [file supp_eov022_New_Microsoft_Office_Word_Document.docx]

**Supplementary Fig. 1.** A) Example on how a representative selection of sequences from each subtype and CRF was achieved. First a maximum likelihood tree from all 12 sequences of this subtype was constructed, and then a selection of one sequence from each of the up to seven most basal lineages from the root was made as a representative sample of the overall known diversity of this particular subtype. B) Schematic illustration of highly divergent sequences. In this example, a, f, g and h (in bold) represent new sequences from a subtype X; the solid circle represents a bootstrap value of >70% while an open circle represents a value of <70%. The arrow shows the basal node of the subtype. In this example, ‘a’ clusters with ‘b’ but with <70% bootstrap support; in addition they form a subtree which also contains ‘c’, ‘d’, ‘e’ and ‘f’ with a <70% bootstrap support; therefore ‘a’ represents an example of a divergent sequence likely clustering near the base of subtype X (it also possible that it clusters nearer the crown). Since the bootstrap support for the branch containing the subtree with ‘c’, ‘d’, ‘e’ and ‘f’ is >70%, ‘d’ is considered to be embedded within the subtype X clade. ‘g’ is located at the base of this subtype. Sequence ‘h’ is a divergent lineage branching outside of subtype X and is likely the extant descendent of a lineage that diverged prior to the diversification of the subtype X MRCA.

**Supplementary Fig. 2.** Recombination analysis of previously described Cameroonian URFs. The multiple genome alignment used to calculate the consensus reference sequences (90% threshold) included the same references as those used for the phylogenetic analyses. The Cameroonian URFs were queried against strains from subtypes A to D, F to H, J, K, CRF01_AE, and CRF02_AG and, in some cases, viruses from lineages that they were most closely related to in the ML tree; the reliability of plot topologies was assessed by bootstrapping with 500 replicates, and a sliding window of 500 bp advancing with 50-bp increments.
