# Supplementary material for: Near full-length HIV type 1M genomic sequences from Cameroon: Evidence of early diverging under-sampled lineages in the country
Source: Evol Med Public Health. 2015 Sep 9;2015(1):254–65. doi: 10.1093/emph/eov022 (PMC4600344; doi:10.1093/emph/eov022)
Supplement: Supplementary Data [file supp_eov022_Supplementary_Figures_for_EMPH_new.pptx]

## Slide 1
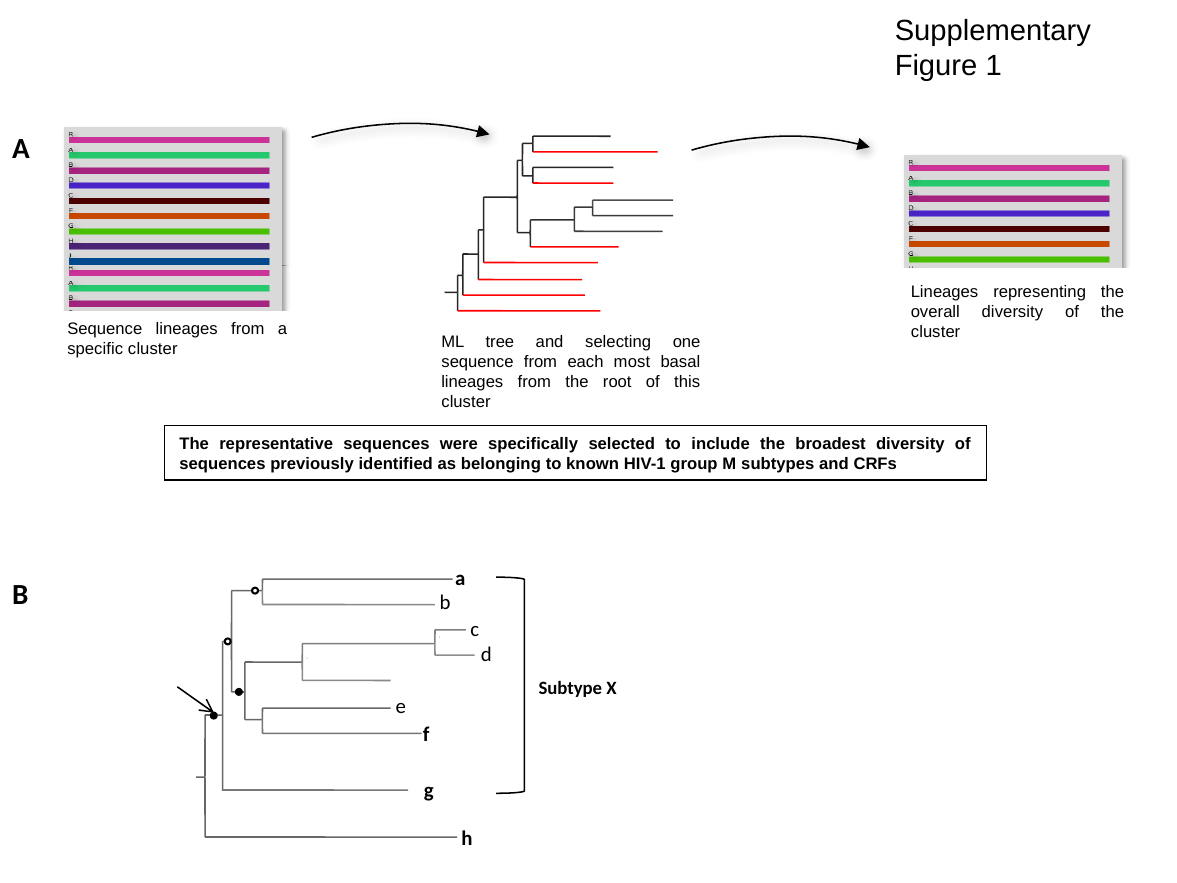

Supplementary Figure 1
A
Lineages representing the overall diversity of the cluster
Sequence lineages from a specific cluster
ML tree and selecting one sequence from each most basal lineages from the root of this cluster
The representative sequences were specifically selected to include the broadest diversity of sequences previously identified as belonging to known HIV-1 group M subtypes and CRFs
a
b
c
d
1
1
e
f
g
h
B
Subtype X

## Slide 2
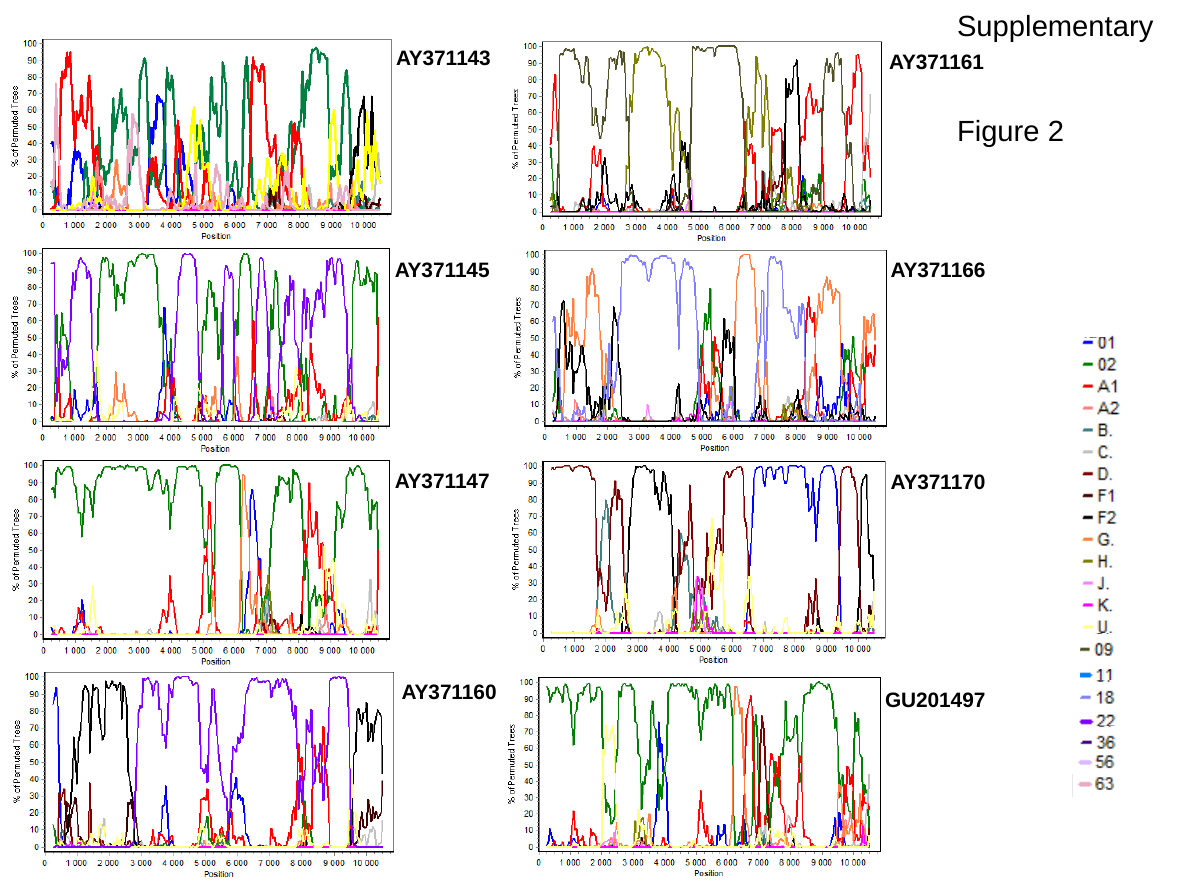

Supplementary 		Figure 2
AY371143
AY371161
AY371145
AY371166
AY371147
AY371170
AY371160
GU201497

## Slide 3
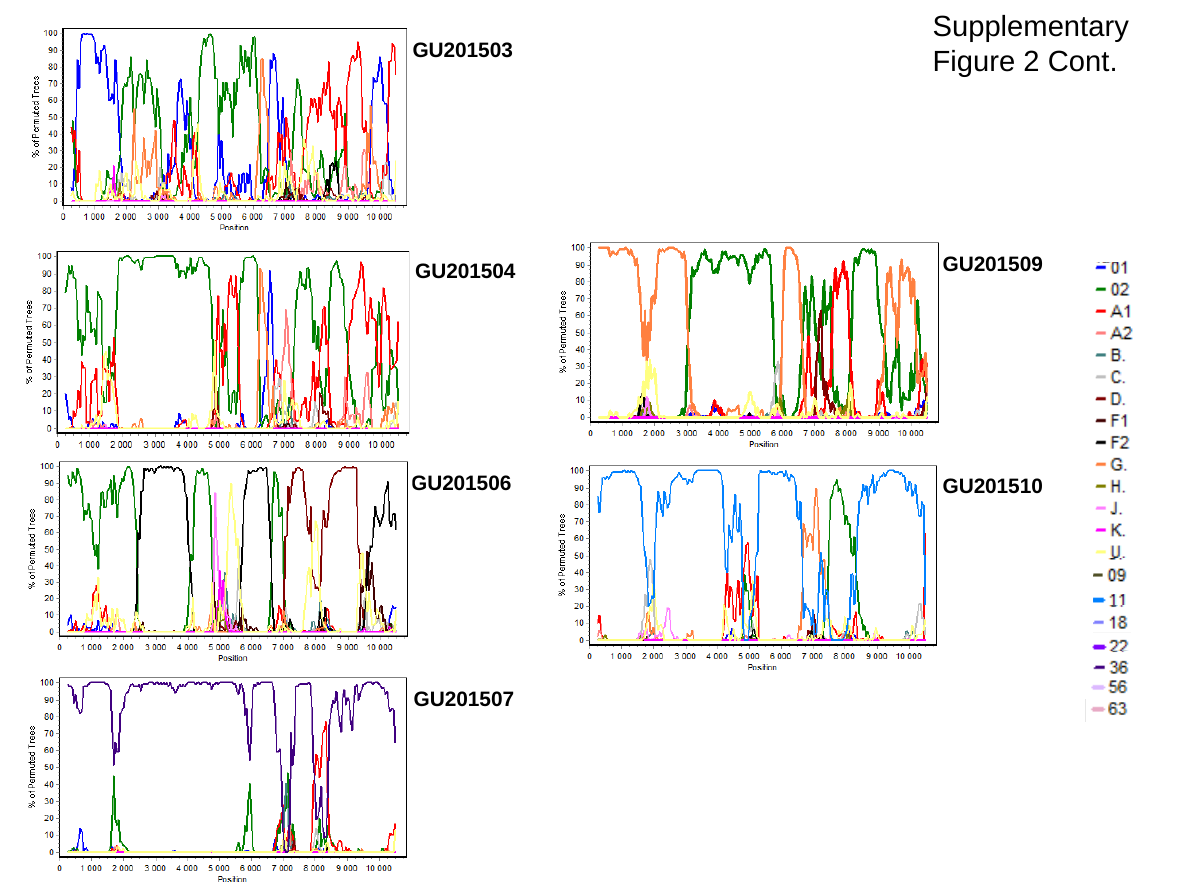

Supplementary Figure 2 Cont.
GU201503
GU201509
GU201504
GU201506
GU201510
GU201507
